# Supplementary material for: Astringency Sensitivity to Tannic Acid: Effect of Ageing and Saliva
Source: Molecules. 2022 Feb 28;27(5):1617. doi: 10.3390/molecules27051617 (PMC8911968; doi:10.3390/molecules27051617)
Supplement: Supplementary file 1 [file molecules-27-01617-s001.zip › molecules-1580401-supplementary.pdf]

Table S1. Description of the tasting solutions used for the training session.

Taste	Salty	Bitter	Sweet	Umami	Sour	Astringent
Component	Sodium chloride (Sigma-Aldrich-France)	Leucine (Dolder Rexim, France)	Sucrose (Sigma-Aldrich, France	Sodium glutamate (Merck, France)	DL-Lactic acid (Fluka, France)	Tannic acid (Sigma-Aldrich, France)
Concentration (g/L)	3	8	15	0.6	2	1.76
